# Stability of serum precipitins to *Aspergillus fumigatus* for the diagnosis of allergic bronchopulmonary aspergillosis

**DOI:** 10.1186/s13223-020-00476-4

**Published:** 2020-09-03

**Authors:** Kevin S. K. Lau, Chantane Yeung, Chris Carlsten

**Affiliations:** 1 Division of Respiratory Medicine, Department of Medicine, University of British Columbia, Vancouver, BC Canada; 2 The Lung Centre, 2775 Laurel St, 7th Floor, Vancouver, BC V5Z 1M9 Canada

**Keywords:** Allergic bronchopulmonary aspergillosis, Aspergillus, Precipitins

## Abstract

**Background:**

Allergic bronchopulmonary aspergillosis (ABPA) reflects hypersensitivity and an exaggerated immune response to *Aspergillus fumigatus*. ABPA typically occurs in individuals with airway diseases such as asthma or cystic fibrosis and is associated with worse outcomes for individuals with these conditions. Each year, physicians across the province of British Columbia submit over 2600 diagnostic testing requests to a centralized location in Vancouver, requiring specimen collection, storage, and shipment from different clinics across the province. Timely and reliable testing of *Aspergillus* precipitins is critical to optimizing diagnosis and management of ABPA. At our centre, we analyzed sample stability in varying storage conditions to provide guidance to those using this routine diagnostic test.

**Methods:**

To determine temperature and time stability, 31 serum specimens positive for *Aspergillus fumigatus* precipitins from routine clinical testing were each aliquoted and incubated at 4 and 37 °C. Samples were repeatedly assayed for precipitins to *Aspergillus fumigatus* via agarose gel double immunodiffusion (AGID) at 7, 14, and 28 days post-incubation.

To determine freeze–thaw stability, 39 serum specimens submitted for routine clinical testing for *Aspergillus* precipitins were randomly selected. Each specimen was aliquoted and stored at 4 or −20 °C. 4 °C samples were maintained at 4 °C while −20 °C samples were split into three groups corresponding to one, two, or three freeze–thaw cycles. −20 °C samples were thawed at room temperature in the morning and then immediately frozen overnight for up to a total of three freeze–thaw cycles.

**Results:**

Regarding temperature and time stability, median stability time was 47 and 34 days at 4 and 37 °C, respectively. The log-rank model indicates no statistically significant difference between the two temperature storage conditions (p = 0.14) with a hazard ratio of 0.61 (95% CI, 0.31–1.2).

In terms of freeze–thaw stability, no indication of serum degradation with regards to *Aspergillus fumigatus* precipitins was found with repeated freeze–thaw cycles as compared to refrigerated storage.

**Conclusions:**

The stability of serum precipitins to *Aspergillus fumigatus* was found to be dependent on time, but not temperature and freeze–thaw cycles. Specimens for *Aspergillus fumigatus* precipitins testing should be shipped at ambient temperature and tested within 2 weeks from collection.

## Background

Allergic bronchopulmonary aspergillosis (ABPA) is a hypersensitivity reaction in response to colonization of the airways with *Aspergillus fumigatus* that occurs in patients with asthma or cystic fibrosis (CF). The global prevalence of ABPA in adults with asthma or CF is estimated to be 2.5% [1] and 18% [2], respectively. There is no definitive test to diagnose ABPA, and there is no consensus on the exact set diagnostic criteria for ABPA. Nonetheless, positive serum precipitins to *Aspergillus fumigatus* is included in most diagnostic guidelines [3], and a positive test result serves as an important indicator in ABPA diagnosis.

Physicians across the province of British Columbia collectively order over 2600 *Aspergillus* precipitins tests annually, requiring specimen collection and storage from various healthcare facilities across the province. These specimens are ultimately shipped to a centralized testing lab in Vancouver. While specimen collection is standardized, storage and shipping conditions can vary across the province. Timely and reliable testing of *Aspergillus* precipitins is critical in the diagnosis and management of ABPA. The aim of the study was to analyze specimen stability in varying conditions to provide provincial specimen handling guidelines for this routine diagnostic test.

## Methods

### Sample collection

Sera was obtained from patients being tested for precipitins to *Aspergillus fumigatus*. 70 serum specimens were collected at various sites belonging to seven health authorities (Vancouver Coastal Health, Fraser Health, Northern Health, Island Health, Interior Health, Provincial Health Services Authority, and Providence Health Care) and two community laboratories (LifeLabs and Valley Medical Laboratories). Whole blood was collected in serum separator tubes, centrifuged as per manufacturer instructions, and stored for 1–10 days prior to shipment to Vancouver General Hospital. Specimen storage and shipment conditions vary by collection site: 4 °C throughout (Vancouver Coastal Health, Providence Health Care, Island Health, Northern Health, Interior Health, Valley Medical Laboratories, and LifeLabs) or initially frozen and shipped at 4 °C (Fraser Health and Provincial Health Services Authority). Upon receipt, specimens were maintained at 4 °C for up to 8 days prior to testing.

### *Aspergillus fumigatus* precipitins via agarose gel double immunodiffusion

0.575% (weight per volume [w/v]) agarose gels were prepared using agarose (Thermo Fisher Scientific, Waltham, USA) dissolved in a solution of 2.9 M NaCl, 82 mM Na_2_HPO_4_, and 9 mM citric acid all purchased from VWR International (Radnor, USA). 1% (w/v) sodium azide (Sigma-Aldrich, St. Louis, USA) was dissolved into the solution once the agarose was fully dissolved.

A Shandon Scientific cutter giving a large central well (13 mm diameter) and six peripheral wells (4 mm diameter) at a distance of 7 mm from the centre was used on a gel with a thickness of 23 mm. Patient sera was dispensed into the centre well and *Aspergillus fumigatus* extract (HollisterStier, Spokane, USA) was dispensed into a peripheral well. Phosphate buffered saline was dispensed into another peripheral well as a negative control.

### Effect of time and temperature on serum precipitins to *Aspergillus*

**Fig. 1:**
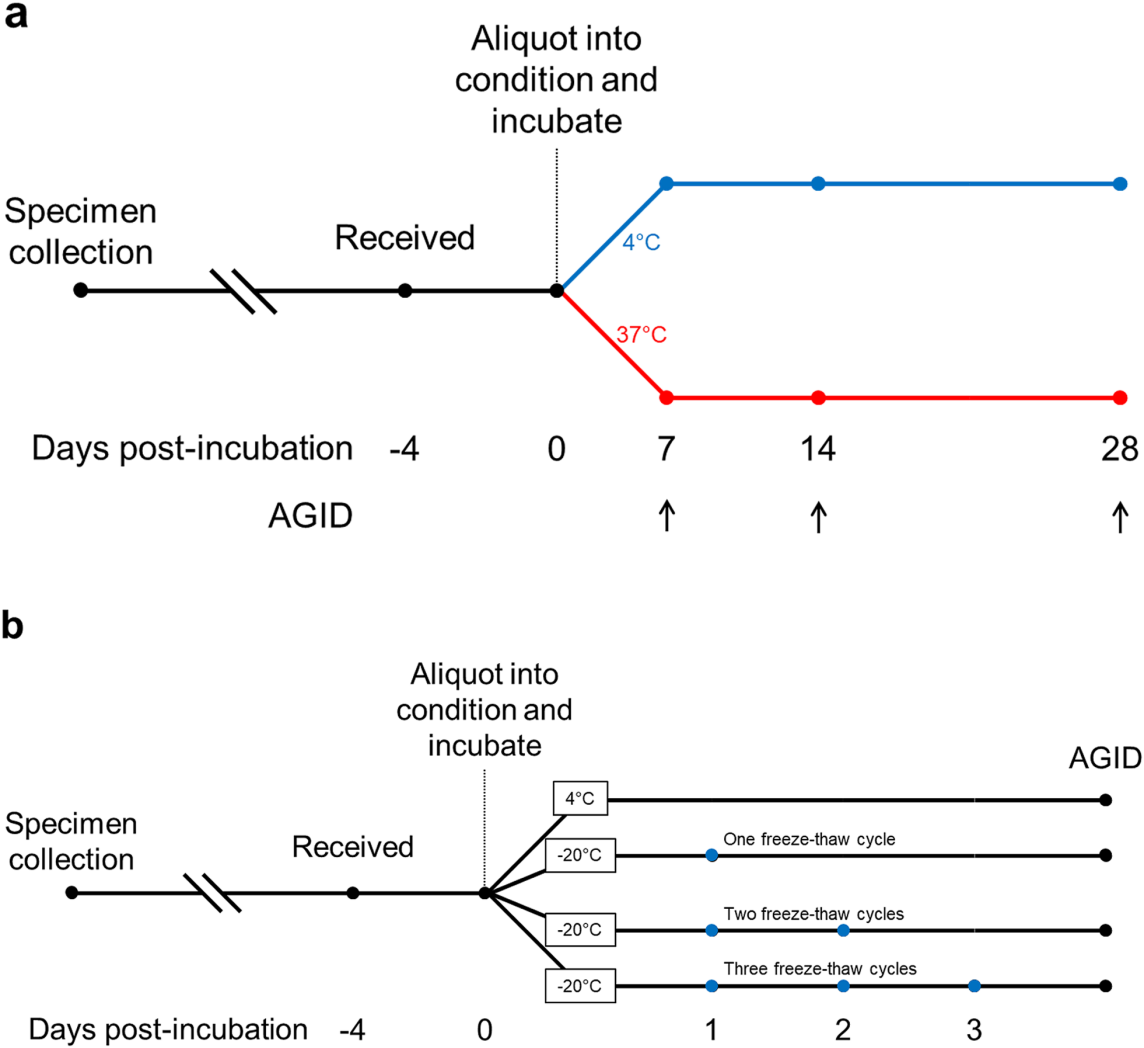
Experimental design. Serum specimens were subjected to differential storage temperatures and repeatedly assayed over time for precipitins to *Aspergillus* via agarose gel immunodiffusion (AGID) (**a**) or subjected to differential numbers of freeze–thaw cycles followed by AGID for *Aspergillus* precipitins (**b**)

31 serum specimens positive for *Aspergillus fumigatus* precipitins from routine clinical testing were each aliquoted and incubated at 4 °C (control) or 37 °C (Fig. 1a). Samples were repeatedly assayed for precipitins to *Aspergillus fumigatus* via AGID at 7, 14, and 28 days post-incubation (Fig. 1a). Stability was regarded as lost when the specimen tested negative for serum precipitins.

All statistical analyses were performed using GraphPad Prism 8 (La Jolla, USA). The temperature groups were compared using the Kaplan–Meier estimate and log-rank test. The hazard ratio was also determined using the same model. The significance threshold was set at p < 0.05.

### Effect of freeze–thaw on serum precipitins to *Aspergillus*

39 specimens out of 152 received for routine *Aspergillus fumigatus* precipitins testing were randomly selected (13 specimens per week over 3 weeks). 4 of the 39 selected specimens originated from facilities that initially froze the specimen and shipped at 4 °C, while the remaining specimens originated from facilities that maintained the specimens at 4 °C throughout. Each specimen was aliquoted and incubated at 4 °C (control) or −20 °C (Fig. 1b). 4 °C samples were maintained at 4 °C; −20 °C samples were thawed at room temperature and immediately refrozen overnight for one, two, or three freeze–thaw cycles (Fig. 1b). Samples were assayed in a randomized order for precipitins to *Aspergillus fumigatus* via AGID. The median time between initial sample collection and assaying was 11 days (mean time was 12 days). Test result agreement with control was used as a proxy for antibody stability.

All statistical analyses were performed using GraphPad Prism 8 and QuickCalcs (La Jolla, USA). Comparison of conditions against control used McNemar’s test. Cohen’s kappa was used to measure chance corrected agreement between conditions. As a guideline for kappa values, we followed Landis and Koch’s recommendations: perfect (κ = 1.00), almost perfect (0.81–0.99), substantial (0.61–0.80), moderate (0.41–0.60), fair (0.21–0.40), slight (0.00–0.20), and poor (< 0.00) [4]. The significance threshold was set at p < 0.05.

## Results

### Effect of time and temperature on serum precipitins to *Aspergillus*

Two specimens from each temperature group were censored at 28 days post-incubation due to insufficient serum available to test all timepoints (Additional file 1). Two specimens that tested as negative at seven days but positive at 14 and 28 days post-incubation were treated as testing positive throughout the study. Four specimens tested negative at 7 and 28 days post-incubation, but positive at 14 days. We interpreted such results at 7, 14, and 28 days post-incubation to be false negatives, true positives, and true negatives, respectively. Specimens are therefore regarded as losing stability by day 28 and were treated as testing positive until 28 days. Seven specimens tested as positive at 7 and 28 days post-incubation, but negative at 14 days. As we are unable to rule out the possibility of false positives on day 28, we opted for a conservative judgement; these specimens were treated as testing positive until 14 days. Three specimens tested negative at 7 and 14 days post-incubation, but positive at 28 days. We opted for a conservative judgement of true negatives at days 7 and 14, and false positives at day 28; these were treated as testing negative throughout the study.

**Fig. 2:**
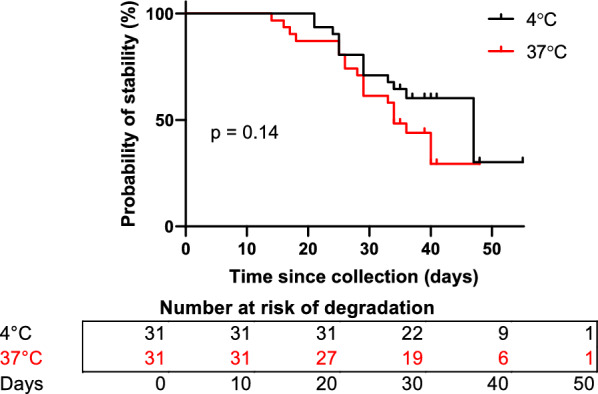
Stability of positive *Aspergillus* precipitins specimens at 4 and 37 °C

Stability of serum precipitins to *Aspergillus* began degrading 14 days from initial specimen collection (Fig. 2). A total of 14 specimens degraded in the 4 °C group and 19 specimens degraded in the 37 °C group. Of the 31 serum specimens that initially tested positive *Aspergillus* precipitins, 31 (100%) and 27 (87%) specimens continued to test positive by 20 days post-specimen collection for the 4 and 37 °C groups, respectively (Fig. 2). By 30 days post-specimen collection, 22 (70%) and 19 (61%) specimens tested positive for the 4 and 37 °C groups, respectively (Fig. 2).

The median stability time of serum precipitins to *Aspergillus* was 47 for the 4 °C (control) group and 34 days for the 37 °C group (Fig. 2). The log-rank test yielded a hazard ratio of 0.61 [95% confidence interval (CI), 0.31–1.21] favouring the 4 °C group, although the Kaplan–Meier curves were found to be not significantly different (p = 0.14).

### Effect of freeze–thaw on serum precipitins to *Aspergillus*

Of the 39 specimens randomly selected for testing in the 4 °C (control) group, 10 tested positive and 29 tested negative for *Aspergillus* precipitins. After one, two, or three freeze–thaw cycles, 13, 11, and 14 specimens tested positive for *Aspergillus* precipitins, respectively, with the remainder testing as negative (Table 1). This corresponds to a false positive rate of 14% (one freeze–thaw), 6.9% (two freeze–thaw), and 17% (three freeze–thaw) for specimens subjected to freeze–thaw cycles. Test specificity was found to be 86% after one freeze–thaw cycle, 93% after two freeze–thaw cycles, and 83% after three freeze–thaw cycles (Table 1). One false negative tracing back to a single specimen was reported across all experimental conditions, resulting in a false positive rate of 10% and a corresponding test sensitivity of 90% (Table 1).

**Table 1:** Diagnostic performance of *Aspergillus* precipitins after one, two, or three freeze–thaw cycles

Diagnostic performance	One freeze–thaw cycle		Two freeze–thaw cycles		Three freeze–thaw cycles	
	% (n)	95% CI	% (n)	95% CI	% (n)	95% CI
Sensitivity	90 (9/10)	60–99	90 (9/10)	60–99	90 (9/10)	60–99
Specificity	86 (25/29)	69–95	93 (27/29)	78–99	83 (24/29)	65–92
Positive predictive value	69 (9/13)	42–87	82 (9/11)	52–97	64 (9/14)	39–84
Negative predictive value	96 (25/26)	81–100	96 (27/28)	82–100	96 (24/25)	80–100

*CI* confidence interval, *n* number

Observed agreement between reference (4 °C) and non-reference (freeze–thaw) conditions were over 85% for all conditions (Table 2). Chance corrected agreement (kappa) ranged from 0.64 (“substantial” agreement) to 0.81 (“almost perfect” agreement) (Table 2). Kappa was not associated with the number of freeze–thaw cycles (p-value range, 0.22–1.00) (Table 2).

**Table 2:** Effect of freeze–thaw on agreement with reference conditions

Number of freeze-thaws cycles	Agreement (%)	κ (95% CI)	*P*
1	87	0.69 (0.45–0.94)	0.37
2	92	0.81 (0.59–1.00)	1.00
3	85	0.64 (0.39–0.90)	0.22

*CI* confidence interval

## Discussion

We are unaware of evidence-based specimen storage and transportation procedures for serum specimens collected to detect precipitins to *Aspergillus* or other proteins within health authorities’ or community laboratories across Canada or elsewhere.

Precipitin reactions in antigen–antibody systems are mediated by both the F(ab’)_2_ and F_c_ fragment, with the F_c_ fragment being the predominant region [5]. Likewise, while both IgG and IgM precipitates in antigen–antibody systems, IgG is the predominant antibody that precipitates due in part to the less exposed F_c_ chain in IgM compared to that of IgG [6]. Taken together, the precipitin reaction is primarily mediated by IgG F_c_.

Our data shows that specimen storage at temperatures above ambient (37 °C) compared to 4 °C does not adversely affect the stability of precipitating antibodies to *Aspergillus*. IgG is considered to be heat-stable relative to other immunoglobulin isotypes. In particular, the F_c_ fragment of IgG was found to be irreversibly denatured at 71 °C [7] whereas heat-labile IgE F_c_ was inactivated at 56 °C [8]. This is in line with previous research where IgG heated to 56 °C for 72 h did not change the intensity of IgG-mediated precipitin arc formation [9]. We do not expect specimens to be subject to these high temperatures in clinical settings, and this data gives us confidence in recommending specimens to be stored and transported at ambient temperatures.

Long-term storage of undiluted serum for up to 3.6 years at −80 °C yielded levels of IgG comparable to fresh sera [10]. Our study shows that serum specimens initially reported as positive for *Aspergillus* precipitins reported as negative over time when stored at 4 °C or higher. Although the increased median specimen stability time was higher for specimens stored at 4 °C, degradation was only found to begin 14 days post-collection. Specimens for clinical testing typically reach our facility 1–10 days post-collection. Taken together, we are confident in recommending specimens to be stored and transported at ambient provided that the specimens reach our facility and are assayed within 14 days post-collection.

We have shown that the number of freeze–thaw cycles does not compromise the stability of precipitating antibodies to *Aspergillus*. While we do not expect repeated freeze–thaw cycles in routine clinical diagnostic settings, serum specimens that have been frozen as a result of repeated serological testing for different diseases without aliquoting are expected to yield results similar to fresh sera. Our results are consistent with previous studies evaluating the stability of IgM and IgG after repeated freeze–thaw cycles via sensitive enzyme immunoassays [10, 11] and adds to the body of evidence that the precipitating characteristics of antibodies (e.g. F_c_ region [5]) against *Aspergillus* have not been functionally altered.

A caveat of our findings is that we assumed our reference test results to be completely accurate, necessarily, given the need for a ‘gold standard’. The flaw of this assumption became apparent as three such positive specimens were repeatedly negative in the time and temperature experiment across both temperature conditions (Additional file 1). To account for this, we would ideally assay specimens repeatedly in order to identify and rectify such aberrations. However, available specimen volumes were limiting in this study, as is typical in this clinical context.

## Conclusion

The stability of serum precipitins to *Aspergillus fumigatus* was found to be dependent on time, but not temperature and/or freeze–thaw cycles. Although multiple freeze–thaw cycles are unlikely to occur in typical clinical settings, this data may have significance to specimens biobanked for research purposes. Specimens for routine clinical testing may therefore be stored and batch transported at ambient temperature within 2 weeks from collection.

## Supplementary information

**Additional file 1.** Summary of results for effect of time and temperature on serum precipitins to Aspergillus. Summary of results showing positive (1) or negative (0) serum precipitins Aspergillus after one, two, or four weeks. Several specimens had insufficient volume (NSQ) to assay at week four. The week where stability was regarded as lost is indicated, with “0” representing specimens that retained stability at week four.

## Data Availability

The datasets used and/or analyzed during the current study are available from the corresponding author on reasonable request.

## Abbreviations

ABPA: Allergic bronchopulmonary aspergillosis
AGID: Agarose gel double immunodiffusion
CF: Cystic fibrosis
CI: Confidence interval
